# Association between phase angle and ECW/TBW ratio with body composition in individuals with central obesity: a cross-sectional study

**DOI:** 10.3389/fnut.2025.1638075

**Published:** 2025-09-30

**Authors:** Agustinus I. Wayan Harimawan, Anak Agung Sagung Mirah Prabandari, Desak Made Wihandani, I. Made Jawi, I. Wayan Weta, Tjokorda Gde Agung Senapathi, Ni Nyoman Ayu Dewi, Luh Putu Ratna Sundari, Christopher Ryalino

**Affiliations:** ^1^Department of Clinical Nutrition, Faculty of Medicine, Udayana University/Prof. IGNG Ngoerah Hospital, Denpasar, Indonesia; ^2^Department of Biochemistry, Faculty of Medicine, Udayana University, Denpasar, Indonesia; ^3^Department of Pharmacology, Faculty of Medicine, Udayana University, Denpasar, Indonesia; ^4^Department of Anesthesiology and Intensive Care, Faculty of Medicine, Udayana University, Denpasar, Indonesia; ^5^Department of Physiology, Faculty of Medicine, Udayana University, Denpasar, Indonesia; ^6^Department of Anesthesiology, University Medical Center Groningen, Groningen, Netherlands

**Keywords:** obesity, abdominal, bioelectrical impedance analysis, phase angle, body composition, extracellular fluid

## Abstract

**Background:**

Central obesity is closely linked with increased metabolic risk, systemic inflammation, and adverse outcomes. Bioelectrical impedance analysis (BIA) provides phase angle (PhA) and extracellular water to total body water (ECW/TBW) ratio—non-invasive biomarkers reflecting cellular integrity and fluid distribution. However, their relationship with detailed body composition in individuals with central obesity remains underexplored. The study aimed to investigate the associations between PhA and ECW/TBW ratio with body composition parameters in individuals with central obesity.

**Methods:**

This cross-sectional study was conducted at a tertiary teaching hospital from December 2024 to February 2025. A total of 741 centrally obese adults (waist circumference ≥90 cm for men and ≥80 cm for women) were assessed using BIA. Parameters analyzed included PhA, ECW/TBW, skeletal muscle index (SMI), fat-free mass index (FFMI), fat mass index (FMI), and visceral adipose tissue (VAT). Statistical analysis involved Pearson correlation and multiple linear regression, stratified by sex.

**Results:**

PhA was positively associated with SMI (*B* = 0.257 in males, B = 0.251 in females; *p* < 0.001) and FFMI (*p* < 0.001), and inversely associated with VAT in males (*B* = −0.082, *p* = 0.017). ECW/TBW ratio was positively associated with FMI and VAT in both sexes (*p* < 0.001) and inversely associated with SMI (*p* = 0.004 in males, *p* < 0.001 in females). Adjusted R^2^ values indicated moderate model fits for muscle-related variables. These findings suggest that lower PhA and higher ECW/TBW ratio are indicative of sarcopenic obesity and fluid imbalance.

**Conclusion:**

PhA and ECW/TBW ratio are associated with distinct components of body composition in central obesity. PhA reflects lean mass and cellular integrity, whereas ECW/TBW ratio captures fluid imbalance and adiposity, though with modest explanatory power. These findings highlight the potential of BIA-derived parameters as complementary tools in nutritional assessment and risk stratification.

## Introduction

Central obesity is a major risk factor for metabolic diseases, including type 2 diabetes, hypertension, and cardiovascular disease (1, 2). Characterized by excessive visceral fat accumulation, it poses greater health risks than general obesity due to its association with systemic inflammation, insulin resistance, and metabolic dysfunction. A recent meta-analysis reported that the global prevalence of obesity was 41.5% (3).

Body composition analysis is essential in obesity assessment, with bioelectrical impedance analysis (BIA) widely used to measure parameters such as total body water (TBW), fat mass, fat-free mass, phase angle (PhA), and the extracellular water to total body water (ECW/TBW) ratio. While dilution techniques (e.g., deuterium or bromide dilution) are regarded as reference standards for TBW and ECW assessment, their high cost, complexity, and invasiveness limit routine clinical and research use (4). BIA offers a non-invasive, rapid, and widely available alternative, allowing for the simultaneous estimation of cellular integrity, fluid distribution, and body composition. Being derived from resistance and reactance, PhA reflects cellular integrity and hydration, with lower values indicating inflammation, muscle loss, or metabolic disturbances common in central obesity (5, 6). Meanwhile, the ECW/TBW ratio indicates fluid distribution and is often associated with fluid retention, inflammation, and metabolic dysfunction, influencing blood pressure, lipid metabolism, and insulin resistance. Moreover, it is also associated with frailty, functional decline, renal dysfunction, and worse outcomes in other populations, making it a clinically relevant marker that deserves investigation in central obesity (7–9).

Despite increasing interest in these BIA-derived indicators, key gaps remain. While PhA shows a strong correlation with fat-free mass, its relationship with fat mass is inconsistent (10). Furthermore, many studies rely on body mass index (BMI) rather than waist circumference to define obesity, limiting their relevance for assessing central adiposity and related metabolic risks (11, 12). Specifically, we emphasize the importance of examining their associations with skeletal muscle and fat indices, as reduced skeletal muscle mass (sarcopenia) is a determinant of frailty and poor outcomes, while excess visceral adiposity contributes to inflammation and metabolic dysfunction. Understanding these inter-relationships provides practical insights for risk stratification and may guide nutritional and fluid management strategies.

To address these gaps, this study aims to evaluate the relationship between PhA and ECW/TBW ratio with body composition in individuals with central obesity. In this study, we focused on PhA and ECW/TBW ratio as the primary indices of interest. Understanding these inter-relationships provides practical insights for risk stratification and may guide nutritional and fluid management strategies. We hypothesized that PhA will correlate positively with indices of muscle mass and cellular integrity, whereas ECW/TBW ratio will associate with markers of adiposity and fluid imbalance.

## Materials and methods

### Study design and settings

This was an observational, cross-sectional study conducted in a tertiary, teaching hospital. The study protocol was approved by the institutional ethics committee (protocol number: 2840/UN14.2.2. VII.14/LT/2024 dated March 11, 2024), and all participants provided written informed consent prior to enrolment. The study was carried out from 1 December 2024 to 28 February 2025. This report adheres to the Strengthening the Reporting of Observational studies in Epidemiology (STROBE) reporting guidelines (13).

### Participants

Participants were recruited from the institution’s employees who met the eligibility criteria and voluntarily agreed to participate. Inclusion criteria were adults aged 18 to 65 years with central obesity, defined by a waist circumference ≥90 cm in males and ≥80 cm in females. Exclusion criteria were pregnancy, presence of metal implants or pacemakers, clinically evident edema or ascites, and ongoing participation in structured weight loss interventions such as bariatric surgery, pharmacological weight management, or intensive dietary programs. These criteria were designed to minimize confounding factors that could affect the accuracy of BIA measurements.

### Variables

The primary variables assessed were PhA and ECW/TBW ratio. Secondary variables included BMI, FFMI, FMI, skeletal muscle index (SMI), and visceral adipose tissue (VAT). These body composition parameters were selected for their relevance to metabolic health, nutritional status, and fluid distribution in central obesity.

### Measurement

All measurements were conducted using the SECA mBCA 514 Medical Body Composition Analyzer (Seca GmbH & Co., Hamburg, Germany), which applies multi-frequency BIA across a range of 1–1,000 kHz. Low-frequency currents predominantly measure extracellular water, whereas higher frequencies penetrate cell membranes to estimate total body water. This multi-frequency approach improves reliability and underpins the calculation of phase angle (derived from reactance and resistance) and the ECW/TBW ratio.

Previous studies have reported the validation of this tool for FFMI, FMI, SMI, and VAT measurements (14–18). The PhA was calculated from reactance and resistance values, representing cell membrane integrity and overall cellular health. The ECW/TBW ratio was used to evaluate extracellular fluid proportion, serving as an indicator of fluid balance and hydration status. SMI and FFMI were used to estimate skeletal muscle mass and fat-free tissue mass, respectively. FMI reflected total fat mass relative to height, and VAT represented intra-abdominal fat distribution, which is closely associated with metabolic risk.

Two trained assessors performed all examinations using a standardized checklist and the manufacturer’s recommended protocol. A protocol simulation with 32 participants, who were not further recruited for this study, was carried out before the study commencement. Inter-rater reliability for BIA measurements was excellent, with ICC (3,1) = 0.92 (95% CI: 0.87–0.96), indicating high reproducibility between assessors. To further minimize inter-rater variability, periodic cross-checks were performed during the study.

To ensure feasibility, participants were scheduled in advance. Participants were instructed to adhere to the following pre-assessment conditions: fasting for 3–4 h prior to the measurement; avoidance of caffeine, alcohol, or energy drinks within the preceding 24 h; and avoidance of strenuous physical activity for at least 12 h. All measurements were performed in the standing position, barefoot, and without any metal accessories. Assessments were conducted between morning and early afternoon to minimize diurnal variations and in a temperature-controlled hospital environment (24–26°C) to minimize environmental influences. The study was conducted between December and February in a tropical climate with minimal seasonal variation, thereby reducing potential confounding due to environmental temperature or seasonality. Each measurement required approximately 10 min, enabling completion of the cohort within the three-month study period. Cross-checks were performed periodically throughout the study to maintain consistency.

### Bias

To minimize measurement bias, all assessments were carried out by trained personnel following strict manufacturer-recommended protocols. The use of a single, validated BIA device and uniform timing of measurements further reduced inter-operator and temporal variability. Exclusion criteria were applied rigorously to eliminate known sources of interference with impedance measurements.

### Study size

The sample size was determined by the total number of eligible and consenting participants available within the study period. Although a formal power calculation was not conducted in advance, the sample size was deemed sufficient for correlation and regression analyses, in line with similar cross-sectional studies assessing body composition and impedance variables.

### Statistical methods

**Figure 1:**
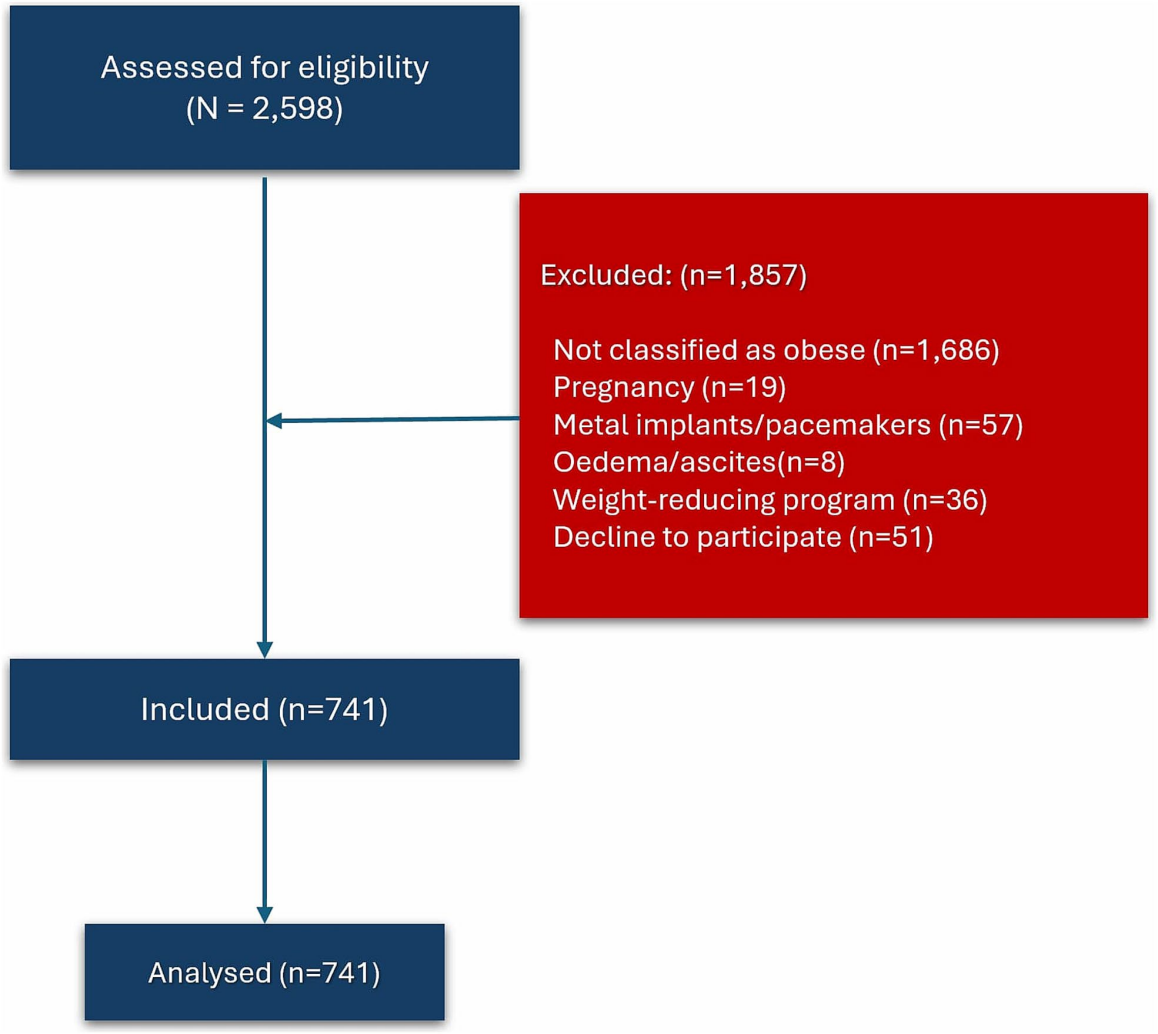
STROBE flow diagram.

**Figure 2:**
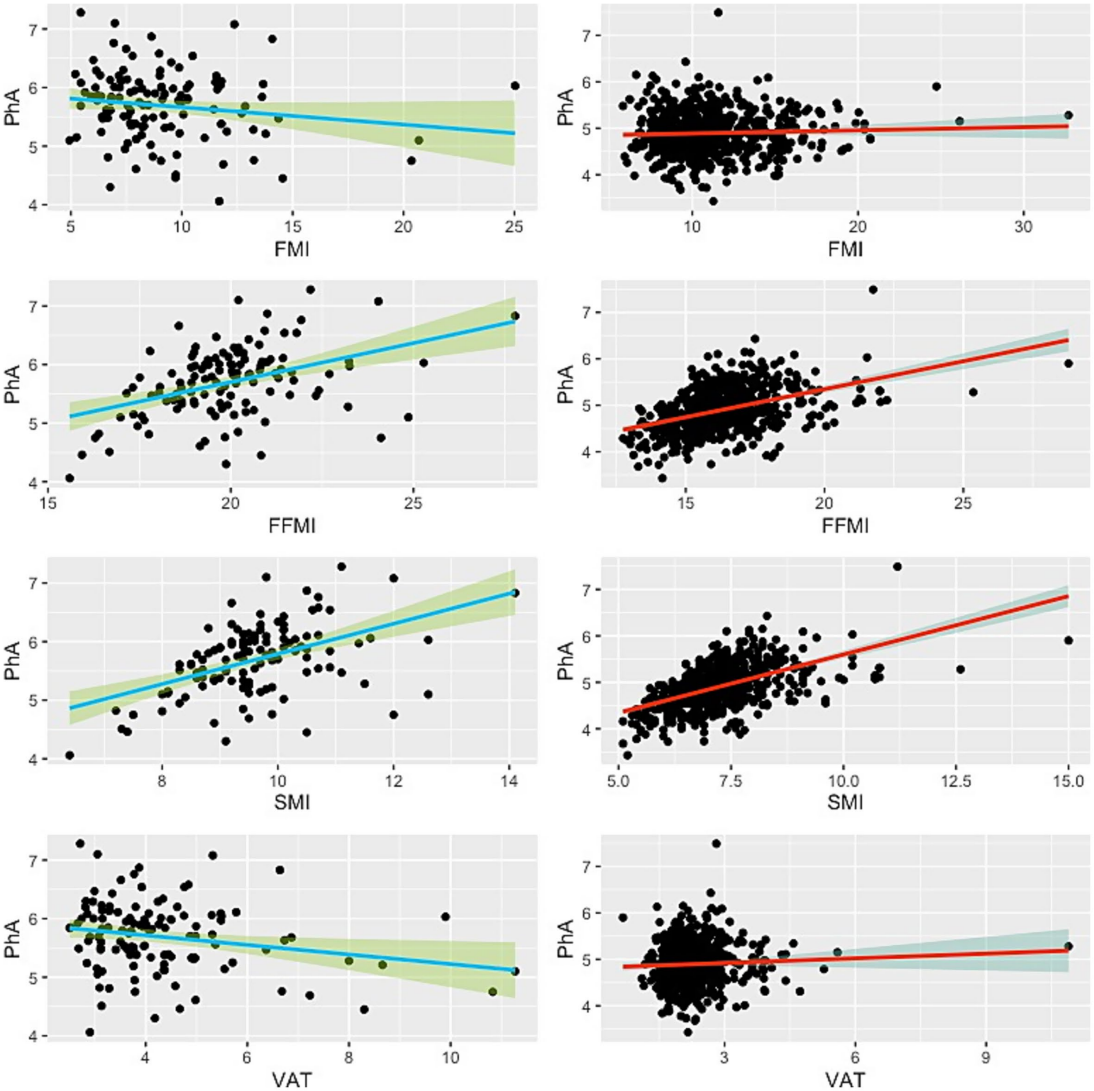
Scatter plot showing the relationship between phase angle and BIA-derived body compositions. Green lines indicate the male subjects, while red lines represent the females. Thick lines illustrate the linear regression line, and the shaded areas denote the 95% confidence interval.

**Figure 3:**
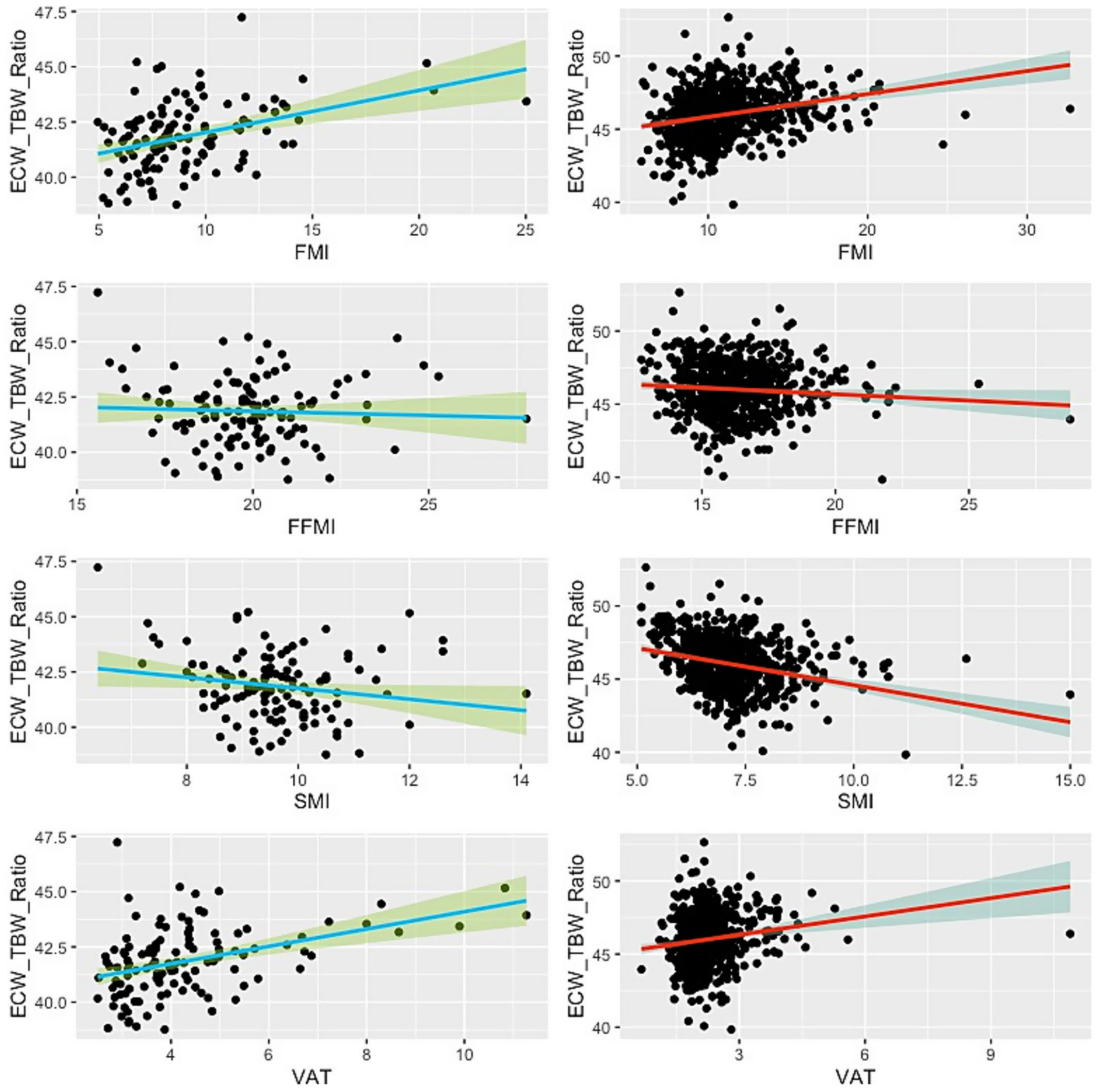
Scatter plot showing the relationship between ECW/TBW ratio and BIA-derived body compositions. Green lines indicate the male subjects, while red lines represent the females. Thick lines illustrate the linear regression line, and the shaded areas denote the 95% confidence interval.

Continuous variables were presented as mean and standard deviation (SD) for normally distributed data, and otherwise as median with interquartile range (IQR). Multiple linear regression analyses were conducted to identify independent associations, reporting unstandardized regression coefficients (B) and 95% confidence intervals (95%CI). Effect sizes were quantified using adjusted R^2^, standardized *β*, and Cohen’s f^2^ values to indicate explained variance. Data analysis was performed using SPSS software version 25 (IBM Corp., Armonk, NY, United States). Figure 1 was designed using Microsoft PowerPoint (Microsoft Inc., Redmond, WA, United States). Figures 2, 3 were created using R for Statistical Computing version 2023.12.1 + 402 (R Core Team, Vienna: Austria) using the ggplot2 (version 3.5.1) package (19). A *p*-value of <0.05 was considered statistically significant.

## Results

### Participant characteristics

Out of 2,598 subjects, a total of 741 were included in this study, comprising 122 males and 619 females (Figure 1). Reasons for exclusions include not classified as obese (*n* = 1,686), pregnancy (*n* = 19), wearing metal implants or pacemakers (*n* = 57), clinically visible oedema or ascites (*n* = 8), following weight-reducing programs (*n* = 36), and decline to participate (*n* = 51).

The mean age of the subjects was 42.91 ± 8.42 years (Table 1), with males being slightly younger on average (40.94 ± 8.74 years) than females (43.29 ± 8.31 years). Males also exhibited higher mean body weight (83.91 ± 14.28 kg vs. 68.24 ± 11.38 kg) and BMI (29.03 ± 4.40 kg/m^2^ vs. 27.32 ± 4.32 kg/m^2^). The SMI, FFMI, and PhA were all higher in males compared to females: SMI (9.61 ± 1.11 vs. 7.19 ± 1.01 kg/m^2^), FFMI (19.91 ± 1.87 vs. 16.24 ± 1.69 kg/m^2^), and PhA (5.69 ± 0.59° vs. 4.89 ± 0.46°). In contrast, females had FMI (11.07 ± 2.98 vs. 9.11 ± 3.07 kg/m^2^) and ECW/TBW ratio (46.01 ± 1.77% vs. 41.85 ± 1.51%) than males. VAT volume was significantly greater in males (4.30 ± 1.57 L) than in females (2.25 ± 0.68 L). Raw resistance and reactance values are provided in the table to allow comparability with population-based reports.

**Table 1:** Characteristics of the study subjects (data presented in mean ± SD).

Variables	Male (*n* = 122)	Female (*n* = 619)	Total (*N* = 741)
Age (years)	40.94 ± 8.74	43.29 ± 8.31	42.91 ± 8.42
Weight (kg)	83.91 ± 14.28	68.24 ± 11.38	70.82 ± 13.24
BMI (kg/m^2^)	29.03 ± 4.40	27.32 ± 4.32	27.61 ± 4.38
Resistance/R (Ω)	537.98 ± 60.40	640.18 ± 72.32	623.35 ± 80.04
Reactance/Xc (Ω)	−53.42 ± 6.74	−54.70 ± 7.09	−54.49 ± 7.05
SMI (kg/m^2^)	9.61 ± 1.11	7.19 ± 1.01	7.59 ± 1.37
FFMI (kg/m^2^)	19.91 ± 1.87	16.24 ± 1.69	16.85 ± 2.20
FMI (kg/m^2^)	9.11 ± 3.07	11.07 ± 2.98	10.76 ± 3.09
VAT (L)	4.30 ± 1.57	2.25 ± 0.68	2.59 ± 1.17
Phase angle (^o^)	5.69 ± 0.59	4.89 ± 0.46	5.03 ± 0.57
ECW/TBW	41.85 ± 1.51	46.01 ± 1.77	45.33 ± 2.32

SD, standard deviation; BMI, body mass index; SMI, skeletal muscle mass index; FFMI, fat-free mass index; FMI, fat mass index; VAT, visceral adipose tissue; ECW, extracellular water; TBW, total body water.

### Association between phase angle and body composition

Linear regression analysis revealed that PhA was significantly positively associated with SMI and FFMI in both sexes (Table 2; Figure 2). For males, the unstandardized regression coefficient (B) for SMI was 0.257 (95% CI: 0.173–0.342, *p* < 0.001; Adjusted *R*^2^ = 0.227), corresponding to a medium-to-large effect size (*f*^2^ = 0.29). For FFMI, B was 0.133 (95% CI: 0.081–0.185, *p* < 0.001; Adjusted *R*^2^ = 0.170; *f*^2^ = 0.20). In females, associations were similarly robust: SMI (*B* = 0.251, 95% CI: 0.251–0.281, *p* < 0.001; Adjusted *R*^2^ = 0.303; *f*^2^ = 0.43) and FFMI (*B* = 0.120, 95% CI: 0.101–0.140, *p* < 0.001; Adjusted *R*^2^ = 0.195; *f*^2^ = 0.24).

**Table 2:** Linear regression model between phase angle and body composition.

Variables	B	95% CI	*P*	Adj R2	β	Cohen’s f^2^
FMI (kg/m^2^)						
Male	−0.030	−0.064, 0.005	0.093	0.015	−0.006	0.015
Female	0.007	−0.005, 0.019	0.272	0.000	0.001	0.000
FFMI (kg/m^2^)						
Male	0.133	0.081, 0.185	<0.001	0.170	0.042	0.205
Female	0.120	0.101, 0.140	<0.001	0.195	0.033	0.242
SMI (kg/m^2^)						
Male	0.257	0.173, 0.342	<0.001	0.227	0.137	0.294
Female	0.251	0.251, 0.281	<0.001	0.303	0.114	0.435
VAT (L)						
Male	−0.082	−0.148, −0.015	0.017	0.039	−0.031	0.041
Female	0.034	−0.020, 0.087	0.216	0.001	0.023	0.001

B, unstandardized B; CI, confidence interval; Adj R^2^, adjusted R^2^; β, standardized B; FMI, fat mass; FFMI, fat-free mass index; SMI, skeletal muscle mass Index; VAT, visceral adipose tissue.

No significant associations were found between PhA and FMI in either sex (*p* = 0.093 in males and *p* = 0.272 in females), with corresponding effect sizes negligible (*f*^2^ ≤ 0.02). A weak but significant inverse association was observed between PhA and VAT in males (*B* = −0.082, 95% CI: −0.148 to −0.015, *p* = 0.017; Adjusted *R*^2^ = 0.039), but the effect size was small (*f*^2^ = 0.04). No significant association was found in females (*p* = 0.216; *f*^2^ ≈ 0).

### Association between ECW/TBW ratio and body composition

The ECW/TBW ratio demonstrated significant positive associations with FMI and VAT in both males and females (Table 3; Figure 3). In males, the ECW/TBW ratio was associated with FMI (*B* = 0.191, 95% CI: 0.108–0.273, *p* < 0.001; Adjusted *R*^2^ = 0.143; *f*^2^ = 0.17) and VAT (*B* = 0.392, 95% CI: 0.234–0.551, *p* < 0.001; Adjusted R^2^ = 0.160; *f*^2^ = 0.19). Among females, associations were weaker: FMI (B = 0.157, 95% CI: 0.111–0.202, *p* < 0.001; Adjusted *R*^2^ = 0.068; *f*^2^ = 0.07) and VAT (*B* = 0.418, 95% CI: 0.215–0.621, *p* < 0.001; Adjusted *R*^2^ = 0.024; *f*^2^ = 0.03).

**Table 3:** Linear regression model between ECW/TBW ratio and body composition.

Variables	*B*	95% CI	*P*	Adj R^2^	β	Cohen’s *f*^2^
FMI (kg/m^2^)						
Male	0.191	0.108, 0.273	<0.001	0.143	0.094	0.167
Female	0.157	0.111, 0.202	<0.001	0.068	0.093	0.073
FFMI (kg/m^2^)						
Male	−0.038	−0.185, 0.107	0.605	−0.006	−0.031	0.000
Female	−0.088	−0.170, −0.005	0.037	0.005	−0.092	0.005
SMI (kg/m^2^)						
Male	−0.247	−0.489, 0.006	0.044	0.025	−0.336	0.026
Female	−0.506	−0.639, −0.374	<0.001	0.082	−0.887	0.089
VAT (L)						
Male	0.392	0.234, 0.551	<0.001	0.160	0.377	0.190
Female	0.418	0.215, 0.621	<0.001	0.024	1.088	0.025

B, unstandardized B; CI, confidence interval; Adj R^2^, adjusted R^2^; β, standardized B; FMI, fat mass; FFMI, fat-free mass index; SMI, skeletal muscle mass Index; VAT, visceral adipose tissue.

In contrast, the ECW/TBW ratio was inversely associated with SMI in both sexes. In males, the effect was modest (*B* = −0.247, 95% CI: −0.489 to −0.006, *p* = 0.044; Adjusted *R*^2^ = 0.025; *f*^2^ = 0.03), while in females the effect was larger but still limited in explanatory power (*B* = −0.506, 95% CI: −0.639 to −0.374, *p* < 0.001; Adjusted *R*^2^ = 0.082; *f*^2^ = 0.09). A weak negative association was also observed between ECW/TBW ratio and FFMI in females (*B* = −0.088, 95% CI: −0.170 to −0.005, *p* = 0.037; Adjusted *R*^2^ = 0.005; *f*^2^ = 0.005), while the association was non-significant in males (*p* = 0.605).

## Discussion

This study investigated the relationship between bioelectrical impedance-derived parameters, specifically PhA and ECW/TBW ratio, and various components of body composition in centrally obese adults. Other BIA-derived measures (SMI, FFMI, FMI, VAT) were included as comparator variables to contextualize the associations and determine whether PhA and ECW/TBW are more closely aligned with muscle-related or fat-related body composition parameters. By including a relatively large sample size and stratifying by sex, we have provided insights into the sex-specific associations between cellular health, fluid distribution, and indices of fat and muscle mass. Men demonstrated higher mean phase angle values and lower ECW/TBW ratios compared with women, consistent with greater muscle mass and intracellular water content. Conversely, women had higher fat mass and ECW/TBW ratios. Although age is known to affect muscle mass, hydration status, and phase angle, our sample was predominantly middle-aged with limited variability. These findings not only contribute to the growing body of knowledge on body composition in obesity but also have particular relevance in the context of clinical nutrition, where fluid balance, nutritional status, and metabolic health are paramount.

Our results demonstrate consistent associations between PhA and indices of lean body mass, particularly SMI and FFMI. This is consistent with prior studies suggesting that PhA reflects cellular integrity, intracellular mass, and metabolic health (5–7). The phase angle, being derived from resistance and reactance, is indicative of the integrity of cell membranes and the relative proportions of intracellular and extracellular water (20). In clinical settings, a higher PhA has been associated with better outcomes, including reduced mortality, shorter length of stay, and improved physical function (21, 22). Our findings reinforce this view, suggesting that PhA could serve as a practical biomarker for identifying individuals with preserved lean tissue mass, even among those with central obesity. Importantly, we emphasize that PhA should not be considered a substitute for direct fat-free mass measurement, but rather a complementary indicator of cellular quality and function that provides prognostic information beyond body mass alone. The higher adjusted R^2^ values in females may reflect differing body composition patterns and hormonal influences on fluid and muscle regulation when compared to males.

The relationship between PhA and FMI was less consistent, with no statistically significant associations in either sex. This aligns with existing literature reporting mixed findings on the relationship between PhA and fat mass (11). Fat mass contributes less to body impedance and may therefore exert limited influence on PhA (12). Furthermore, adipose tissue is less metabolically active and electrically conductive than muscle (23), which may explain the weak correlation. The weak and inverse association observed between PhA and VAT in males, though modest in effect size, suggests that higher VAT may compromise cellular function or be associated with greater inflammatory load (24, 25), both of which could lower PhA values. This association was not significant in females, which may reflect differences in fat distribution and hormonal regulation. For instance, men are more prone to visceral fat accumulation, whereas women tend to accumulate more subcutaneous fat, a pattern influenced by sex hormones such as estrogen and testosterone. These physiological and hormonal differences may partly explain why certain associations, such as the PhA–VAT relationship, were observed in men but not women.

The ECW/TBW ratio, on the other hand, showed significant positive associations with fat-related parameters (FMI and VAT) and negative associations with muscle-related parameters (SMI and FFMI), particularly in females. The ECW/TBW ratio is an important marker of fluid distribution and cellular hydration status (26, 27). Elevated ECW/TBW ratios have been associated with inflammation, fluid overload, and worse clinical outcomes in various patient populations, including those in intensive care units (8–10, 26, 28–30). The positive relationship between ECW/TBW and fat mass components may reflect the pro-inflammatory state of obesity, which can lead to fluid shifts from the intracellular to extracellular compartments (31, 32).

In our study, however, these associations explained only a small proportion of variance (adjusted R^2^ between 2 and 8%), meaning that they should be interpreted as modest in strength despite their statistical significance. Nevertheless, these associations are consistent with established pathophysiological mechanisms: excess adiposity and inflammation can lead to extracellular fluid expansion, while muscle mass contributes to intracellular water and buffers against fluid imbalance (33). Therefore, preserving muscle mass in obese individuals may help mitigate the risk of fluid derangement and its complications (34, 35). Notably, ECW/TBW ratio demonstrated medium effect sizes for fat indices and VAT in males (*f*^2^ = 0.17–0.19), suggesting modest clinical relevance in this subgroup. These findings indicate that while the large sample size contributed to detecting statistical significance, only a subset of the associations are of medium-to-large strength and thus potentially meaningful for clinical application.

From a clinical standpoint, these results highlight the possibility of incorporating PhA and ECW/TBW assessments into routine body composition analysis for obese patients. These indices offer rapid, non-invasive means of evaluating cellular health, hydration status, and metabolic reserve—parameters that are crucial for decision-making in nutrition support, fluid therapy, and rehabilitation. For instance, a declining PhA or rising ECW/TBW ratio during hospitalization may signal deteriorating nutritional status or emerging fluid imbalance, warranting early intervention (36, 37). Nevertheless, their role should be seen as complementary rather than substitutive. PhA provides insight into cellular integrity, while ECW/TBW captures fluid distribution, both extending the interpretative value of standard fat and muscle indices derived from BIA.

The integration of PhA and ECW/TBW assessments into clinical practice may offer promising avenues for personalized nutrition and fluid management. These parameters could complement traditional metrics such as BMI and waist circumference by providing insight into tissue quality and fluid dynamics. Early identification of patients at risk for sarcopenia, inflammation, or fluid overload can enable targeted interventions that improve recovery, reduce complications, and shorten length of stay. Furthermore, BIA-derived metrics could support decision-making in preoperative assessments, especially in bariatric or abdominal surgeries where fluid shifts and nutritional status are critical determinants of postoperative outcomes.

This study has several limitations that should be acknowledged. First, its cross-sectional design precludes any causal inferences between body composition parameters and BIA-derived indices. Longitudinal studies are necessary to determine whether changes in PhA or ECW/TBW predict clinical outcomes over time. Second, concerns may arise because PhA, ECW/TBW ratio, and skeletal muscle indices are all derived from BIA, raising the possibility of collinearity. However, these parameters represent distinct physiological domains: PhA reflects membrane integrity, ECW/TBW indicates extracellular-to-intracellular water distribution, and muscle indices quantify contractile tissue. Third, although BIA is a validated method for body composition analysis, its accuracy can be influenced by hydration status, recent food or fluid intake, and physical activity levels. Additionally, the heavily skewed population toward females, which reflects the demographic distribution of the hospital workforce from which the participants were recruited, limited the statistical power of male subgroup analyses. Sex-specific findings in men should therefore be interpreted with caution. Furthermore, the adjusted R^2^ values for several models, especially those involving ECW/TBW, were low, indicating that the predictors explained only a small portion of outcome variance. Thus, these findings are associative and not predictive, and their clinical significance should be interpreted carefully. It should also be noted that phase angle is universally defined as the arctangent of reactance and resistance, and thus comparable across devices and studies. In contrast, the ECW/TBW ratio is derived from device-specific algorithms that translate impedance values into fluid compartment estimates. This algorithm dependence may introduce variability between instruments, which limits comparability across studies and should be considered when interpreting our findings. Finally, while our strict adherence to consistent timing, fasting state, and pre-assessment conditions helped minimize variability, we recognize that standing vs. supine positioning could introduce subtle differences in BIA-derived measurements.

## Conclusion

In conclusion, this study demonstrates that phase angle and ECW/TBW ratio are associated with distinct components of body composition in centrally obese adults, although the strength of these associations varies. Phase angle showed medium-to-large effects for skeletal muscle and fat-free mass indices, supporting its role as a complementary marker of lean tissue quality and cellular integrity. By contrast, associations between ECW/TBW ratio and muscle indices were modest, while medium effects were observed for fat indices and VAT, particularly in men. Taken together, these results suggest that BIA-derived parameters provide complementary insights into cellular health and fluid balance, but should not be used as substitutes for direct measurements of body composition.

Future research should aim to validate these findings in prospective cohorts, including patients admitted to critical care units, surgical wards, or undergoing nutritional rehabilitation. Interventional studies exploring the impact of tailored nutrition, resistance training, or fluid restriction on PhA and ECW/TBW could further elucidate their clinical utility. Moreover, comparisons with the normal-weight and overweight populations would provide additional context. Also, future studies should incorporate objective or validated measures of physical activity to better account for lifestyle influences on body composition and impedance-derived indices. Additionally, incorporating inflammatory markers, renal function parameters, and functional outcomes (e.g., handgrip strength, length of ICU stay) would enhance the understanding of these associations.

## Data Availability

The raw data supporting the conclusions of this article will be made available by the authors, without undue reservation.
